# Extracellular DNA Is Essential for Maintaining *Bordetella* Biofilm Integrity on Abiotic Surfaces and in the Upper Respiratory Tract of Mice

**DOI:** 10.1371/journal.pone.0016861

**Published:** 2011-02-11

**Authors:** Matt S. Conover, Meenu Mishra, Rajendar Deora

**Affiliations:** 1 Program in Molecular Genetics, Wake Forest University Health Sciences, Winston-Salem, North Carolina, United States of America; 2 Department of Microbiology and Immunology, Wake Forest University Health Sciences, Winston-Salem, North Carolina, United States of America; Institut de Pharmacologie et de Biologie Structurale, France

## Abstract

Bacteria form complex and highly elaborate surface adherent communities known as biofilms which are held together by a self-produced extracellular matrix. We have previously shown that by adopting a biofilm mode of existence *in vivo*, the Gram negative bacterial pathogens *Bordetella bronchiseptica* and *Bordetella pertussis* are able to efficiently colonize and persist in the mammalian respiratory tract. In general, the bacterial biofilm matrix includes polysaccharides, proteins and extracellular DNA (eDNA). In this report, we investigated the function of DNA in *Bordetella* biofilm development. We show that DNA is a significant component of *Bordetella* biofilm matrix. Addition of DNase I at the initiation of biofilm growth inhibited biofilm formation. Treatment of pre-established mature biofilms formed under both static and flow conditions with DNase I led to a disruption of the biofilm biomass. We next investigated whether eDNA played a role in biofilms formed in the mouse respiratory tract. DNase I treatment of nasal biofilms caused considerable dissolution of the biofilm biomass. In conclusion, these results suggest that eDNA is a crucial structural matrix component of both *in vitro* and *in vivo* formed *Bordetella* biofilms. This is the first evidence for the ability of DNase I to disrupt bacterial biofilms formed on host organs.

## Introduction

The genus *Bordetella* currently consists of nine species of Gram negative bacteria. Some members of this genus are known mammalian and avian pathogens that colonize the respiratory tracts of humans, animals and birds. *B. pertussis* and some strains of *B. parapertussis* are the causative agents of whooping cough in humans, while *B. bronchiseptica* causes multiple respiratory syndromes and diseases in a wide variety of animal species, including dogs, pigs, cats, rabbits and rats. *B. avium* infects commercially grown turkeys as well as wild and domesticated birds [1], [2], [3].

A hallmark of *B. bronchiseptica* and *B. avium* infections is long-term to life-long asymptomatic carriage. Although vaccination considerably decreases mortality and severity of the respiratory disease, *B. bronchiseptica* and *B. avium* continue to circulate and persist in mammalian and avian species. *B. bronchiseptica* is frequently isolated from the nasal cavities of vaccinated animals suggesting that vaccines fail to protect animals from infections [4]. Similarly, despite excellent vaccine coverage, pertussis remains endemic in the USA and many European countries. Outbreaks of pertussis are observed frequently. It is becoming clear that the current pertussis vaccines, although effective against severe symptoms of the disease, do not prevent prolonged colonization. *B. pertussis* continues to circulate by residing mainly in the nasopharynx of adolescents and adults, resulting in asymptomatic or milder infections [5], [6], [7].

Despite enhanced awareness of the need for increased and efficient detection [8], a large number of adult pertussis cases often remain undiagnosed [6], [9]. Infected individuals silently harbour the pathogen, resulting in heightened transmission risk to susceptible children [10], [11]. Intra-familial and other modes of person-person pertussis transmission have been documented [10], [12]. In a recent population-based study of families having an infant diagnosed with pertussis, 53% of the household contacts had laboratory-confirmed pertussis. Strikingly, in 60% of the households, the source of transmission to infants was clearly established to be one of the family members [13].

One proposed hypothesis to explain the survival and continued persistence of *Bordetella Spp.* in the mammalian nasopharynx is that these organisms form surface-adherent communities known as biofilms [14], [15]. Recent studies from our laboratory and others have supported this hypothesis by demonstrating that both *B. pertussis* and *B. bronchiseptica* are capable of forming biofilms on abiotic surfaces [16], [17], [18], [19] and in the mouse respiratory tract [14], [15]. The ability to form biofilms in mice suggests a role for this mode of existence during human infections. In this context, clusters and tangles (reminiscent of biofilms) of *B. pertussis* adherent to ciliated cells in explant cultures and tissue biopsies of pertussis patients have been demonstrated [20], [21], [22].

Biofilms are defined as a community of surface-adherent bacteria encased in a self-produced polymeric matrix that holds the cells together. Limitations of oxygen within the biofilm matrix, altered metabolic rate of the surface-adherent organisms combined with the function of the matrix as a physical barrier results in biofilm cells becoming resistance to killing by host defenses, antimicrobial compounds and surfactants [23], [24]. While the composition of biofilm matrices varies depending upon the bacterial species, growth media or the environmental conditions, it is often composed of a polysaccharide biopolymer along with proteins and extracellular DNA (eDNA) [25], [26], [27]. eDNA has now emerged as one of the major components the biofilm matrix of many bacteria and has been shown to perform diverse functions in promoting the biofilm mode of existence [27], .

Previous studies from our laboratory and others have clearly established that, like some bacterial pathogens, *Bordetella* biofilm development is also mutifactorial [24], [26]. We have shown that the exopolysaccharide Bps is a component of the biofilm matrix and is essential for maintaining the biofilm architecture in both *B. bronchiseptica* and *B. pertussis* [14], [15], [17]. Others have examined mutant strains containing deletions in outermembrane proteins that show biofilm formation in the animal pathogen *B. bronchiseptica* is protein-mediated [18]. In this report, we demonstrate that DNA is a significant component of the *Bordetella* biofilm matrix. We show that DNase I not only led to inhibition of biofilm growth, but also disrupted established mature biofilms formed under both static and continuous flow conditions. These results provide strong evidence for the crucial function of eDNA in maintaining the integrity of laboratory biofilms formed on artificial surfaces.

Despite the wealth of *in vitro* data on the different bacterial biofilm developmental programs and the mechanisms by which many bacterial factors contribute to these processes, large gaps exist in the research on biofilms formed on host organs. Very few animal models are able to mimic the characteristics of biofilms formed *in vitro*. We have recently established an *in vivo* model of *Bordetella* biofilms in the mouse respiratory tract [14], [15]. One of the strengths of this model is that the *Bordetella* microcolonies attached to the nasal epithelium are surrounded by an extracellular matrix composed of the Bps polysaccharide, thereby satisfying the definition of *in vivo* biofilms [23], [33]. To examine if DNA contributed to the structural stability of these nasal biofilms, we treated nasal septa from mice infected with *B. bronchiseptica* and *B. pertussis* with DNase I. Immunofluorescence and scanning electron microscopic examination revealed that this treatment resulted in considerable dissolution of nasopharyngeal biofilms. These results suggest that DNA promotes the stability and development of *Bordetell*a biofilms in the upper respiratory tract.

## Materials and Methods

### Bacterial strains and culture conditions

Wild type strain (RB50) of *B. bronchiseptica* was grown in Stainer-Scholte (SS) broth [57]. For culturing *B. pertussis* strain (Bp536), the SS medium was supplemented with heptakis (6-di-O-methyl-β-cyclodextrin) [16]. For culturing strains harboring the pTac-Gfp plasmid, the SS medium was supplemented with 50 µg/ml of chloramphenicol [17]. All strains were maintained on Bordet-Gengou (BG) agar supplemented with 7.5% defibrinated sheep blood and streptomycin (50µg/ml).

### Growth and treatment with DNase I of static biofilms

Biofilms were cultivated in microtitre plates at 37°C as described previously [17]. Briefly overnight grown culture of *B. bronchiseptica* was inoculated in the wells of a 96 well microtitre plate at OD_600_ of 0.05 followed by addition of either DNase I reaction buffer (10mM Tris-HCl, pH 7.5, 50% glycerol, 10mM MgCl_2_), or with DNase I (40 Kuntz units/ml)(Sigma or Promega) resuspended in the DNase I reaction buffer. A time course of biofilm formation was performed over 48h under static conditions. At each time point, non attached planktonic bacteria were removed and the plate was rinsed thoroughly. The biofilms were stained with 100µl of 0.1% crystal violet solution by incubating at room temperature for 30 min. After washing, the attached crystal violet was solubilized in 200 µl of 95% ethanol and transferred to a new polystyrene plate and absorbance was measured at 540nm. A similar procedure was employed for *B. pertussis* but with some modifications. The biofilms were grown in 12 well tissue culture plates and these plates were inoculated with *B. pertussis* cultures grown for 3–4 days [15].

### Reversibility of DNase I inhibition

*B. bronchiseptica* biofilms were inoculated as described above with DNase I or media alone. After 24h, the media was removed from one set of six wells containing DNase I as well as the mock wells and replaced with fresh media. The original media and DNase I was left undisturbed in an additional set of six wells. All samples were then incubated at 37°C for an additional 24h before processing with crystal violet as described above.

### DNase I stability assays

DNase I from both Sigma and Promega were incubated at 37°C for 48h in their appropriate reaction buffer at a concentration of 100 Kuntz units/ml. Following the incubation, approximately 1 µg of genomic DNA from RB50 was added. The reactions were continued for 3h at 37°C. Samples were then run on a 1% agarose gel and stained with ethidium bromide for visualization of the DNA.

### Growth and treatment with DNase I of pre-existing static biofilms

*B. bronchiseptica* cultures were inoculated into 96 well plates in a 100µl volume at an OD_600_ of 0.05. Biofilms were then allowed to form for 48h at 37°C under static conditions. After 2 days, the biofilms were washed once with sterile PBS before the addition of either 100µl of sterile PBS, PBS with reaction buffer, PBS with 40 Kuntz units/ml of DNase I, PBS with 40 Kuntz units/ml of heat inactivated DNase I, or PBS with reaction buffer and DNase I for 2h at 37°C. All wells were then washed 3 times and then treated for crystal violet staining as described above.

Mid log phase (OD_600_∼0.7–1.0) grown cultures of *B. bronchiseptica* and *B. pertussis* carrying the pTac-Gfp construct were inoculated in two chambered coverslips and cultured in SS broth with chloramphenicol. 12mm glass coverslips were partially submerged in the culture and allowed to incubate for 48h with *B. bronchiseptica* or 96h with *B. pertussis* at 37°C under static conditions. Biofilms formed at the air liquid interface of the coverslips were gently rinsed with PBS to remove any unattached bacteria, followed by incubation with DNase I for 30 min or 90 min at 37°C. Coverslips were gently rinsed with PBS and mounted with ProLong gold antifade reagent (Invitrogen), followed by visualization using a Zeiss LSM 510 confocal scanning laser microscope. Coverslips were incubated with PBS as a negative control and treated in a similar manner.

### Flow cell biofilms and DNase I treatment

Biofilms were grown in three chambered flow cells (Stovall), which were aseptically inoculated with 200µl of mid log phase culture (OD_600_∼0.7–1.0) of Gfp-tagged RB50 cells using sterile 25 5/8 gauge needles, followed by incubation at 37°C for 2h. Subsequently flow of the SS media was initiated at a constant rate of 0.3ml/min. At various time points (6, 72 and 120h), flow was stopped and the biofilms were treated with 66 Kuntz units/ml of DNase I supplemented with 5mM MgCl_2_ for 90 min. After this incubation, the channels were rinsed gently by resuming the flow at the previous rate. Biofilm growth and accumulation of extracellular DNA were visualized under 63× water objective by CSLM. Extracellular DNA in the biofilm was stained with 2µM DDAO [7-hydroxy-9H-(1, 3-dichloro-9, 9 dimethylacridin-2-one)] for 30 min at 37°C.

### Ethics statement

Animal husbandry and experimental procedures were performed in accordance with Public Health Service policy and the recommendations of the Association for Assessment and Accreditation of Laboratory Animal Care and approved by the Wake Forest University Health Sciences Institutional Animal Care and Use Committee (protocol #s A09-146, A06-153, A09-024).

### *In vivo* biofilm formation on the mouse nasal septum, DNase I treatment and immunofluorescence staining

Five to six week-old female C57BL/6 mice (Jackson Laboratory) were partially anesthetized using isoflurane (Butler) and infected intranasally with ∼5×10^5^ cfus of RB50 or Bp536 in a 50µl droplet. At 15 and 19 days after inoculation with RB50 or Bp536, the nasal septa were excised and cut into two equal parts. One half was incubated with PBS while the other half was treated with 100 Kuntz unit/ml of DNase I supplemented with 5mM MgCl_2_ for 90 min at 37°C. Both nasal sections were then briefly washed with PBS before fixation with 2.5% glutaraldehyde.

Nasal septa were stained as described previously [58], Briefly, fixed mouse nasal septa were rinsed with PBS and blocked with 5% normal donkey serum in PBS for 30 min. Immunochemical staining of RB50 was performed using a polyclonal mouse sera raised against a Bvg^+^ phase locked derivative of RB50. For *B. pertussis* biofilms, polyclonal mouse sera raised against Bp536 was used. Both sera were used at a 1∶1000 dilution in PBS. The nasal septa were incubated at room temperature for 2h. After multiple washes with PBS, nasal septa were incubated with a 1∶2000 dilution of goat anti-mouse antibody conjugated to Alexa fluor 488 for 1.5h. Samples were washed with PBS and permeabilized with 0.1% Triton X-100 for 5 min, followed by staining with 1∶40 dilution of phalloidin conjugated with Alexa fluor 633 for 30 min for visualization of eukaryotic F actin. Samples were washed again with PBS and mounted using ProLong Antifade gold reagent (Invitrogen) according to manufacturer's instructions, and stored in the dark to dry. The slides were viewed on a Zeiss 510 confocal laser scanning microscope.

### Scanning Electron microscopy (SEM)

Nasal septa harvested from infected or naïve mice were treated with either DNase I (as described above) or PBS as a negative control. Samples were gently rinsed with PBS after treatment followed by fixation with 2.5% glutaraldehyde for 1h followed by routine processing for SEM as described previously [16].

### Statistical analysis

All statistics were performed using the Student's *t*-test and were determined to be significant if P<0.05.

## Results

### The *Bordetella* biofilm matrix contains DNA

**Figure 1:**
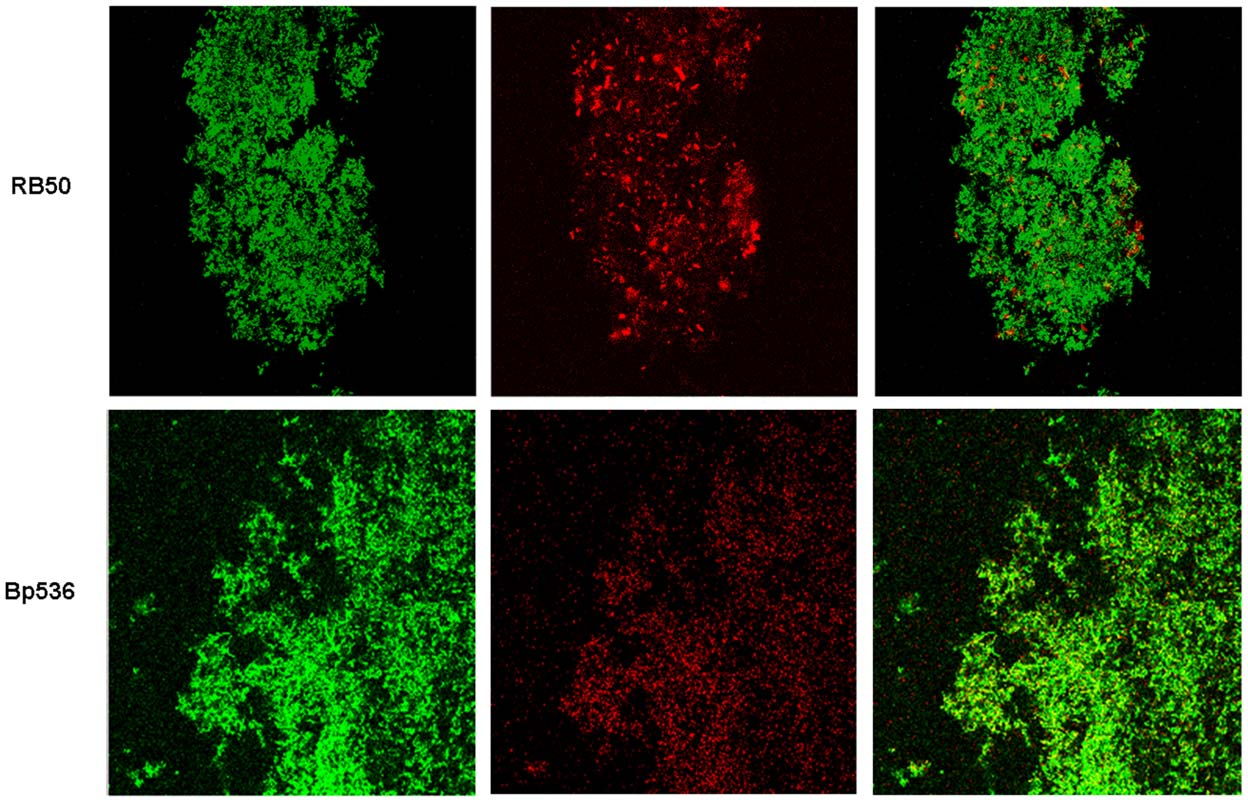
Bordetella biofilms contain eDNA. Three day old biofilms of *B. bronchiseptica* strain RB50 (upper panels) and *B. pertussis* strain Bp536 (lower panels) were stained with DDAO. CLSM images of live GFP expressing cells (green) and DDAO stained eDNA (diffuse red) or dead cells (punctuate red) are shown. Yellow appearance indicates the presence of both live cells and eDNA. The images shown are representative of three independent experiments.

In our ongoing studies focused towards identifying the constituents of the *Bordetella* biofilm matrix, we determined whether DNA is a component of the biofilm matrix. GFP expressing cells were inoculated in chambered coverslips and allowed to form biofilms under dynamic flow conditions. After 72h of flow, the biofilms were stained with DDAO which binds to extracellular DNA (eDNA) or DNA inside the cells that have compromised membranes [34]. The results showed that biofilms formed by the wild type strain of *B. bronchiseptica*, RB50 (Fig. 1, top panels) were mainly composed of cells which were not stained by DDAO, and thus have impermeable membranes. In addition to the green-staining live cells, bright red-staining dead cells were observed along with a more diffuse weaker red stain which is indicative of eDNA.

Similar to *B. bronchiseptica*, *B. pertussis* biofilms exhibited both bright red-staining cells and a comparatively more diffuse weaker red stain interspersed among the GFP-expressing live cells (Fig. 1, bottom panels). This staining pattern is similar to eDNA detected in other organisms such as *Enterococcus*, *Listeria*, and *Staphylococcus* [35], [36], [37]. Taken together, these results suggest that DNA is a component of biofilms formed by both *B. bronchiseptica* and *B. pertussis*.

### Extracellular DNA is required for biofilm formation and stability

**Figure 2:**
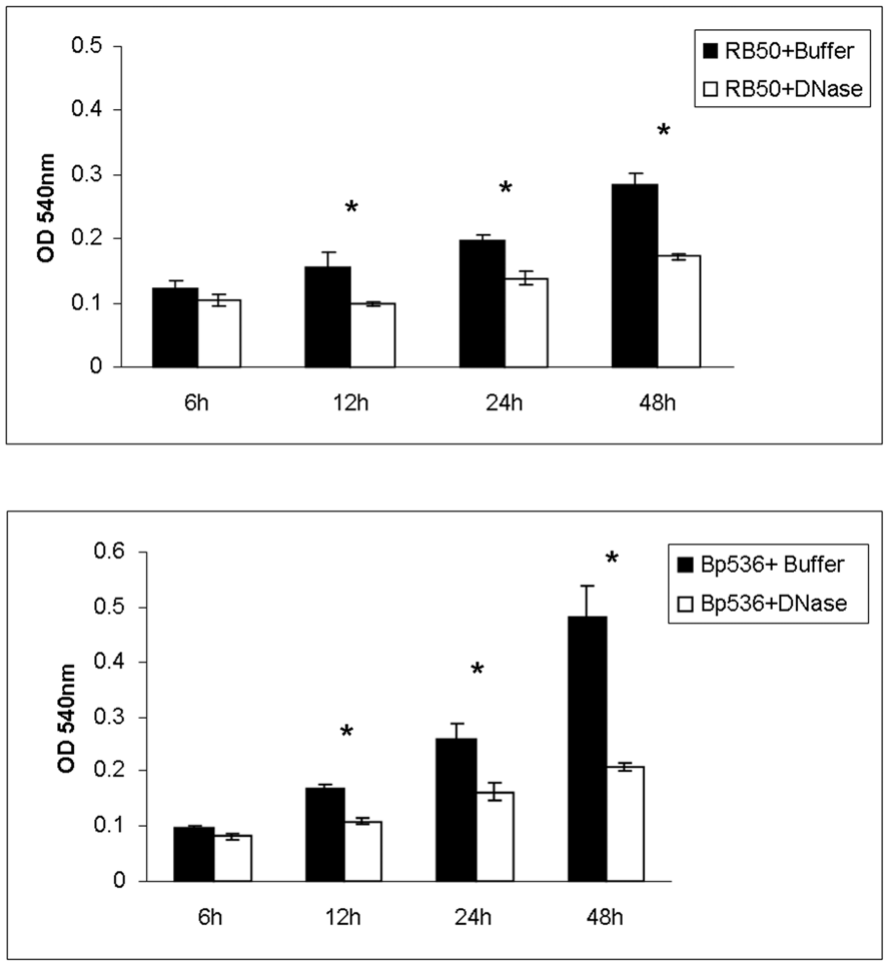
DNase I inhibits *Bordetella* biofilm formation. The indicated strains were grown in 96 well microtitre plates for RB50 or 12 well plates for Bp536 for designated time points in SS medium supplemented with either DNase I resuspended in the reaction buffer or the reaction buffer alone. Wells were rinsed and stained with crystal violet followed by quantification of bound crystal violet as described in Materials and Methods. Each data point is the average of six wells, and error bars indicate the standard deviation. Representative data from one of at least three independent experiments are shown. Asterisks designate a value of *P*<0.05 (students *t*-test).

We hypothesized that by acting as a structural component, DNA promotes biofilm stability. If this is the case, we reasoned that removal of extracellular DNA from the biofilm matrix, by treatment with DNase I, will lead to a reduction in the formation of biofilms. Thus, we examined the ability of DNase I to inhibit biofilm formation of both *B. bronchiseptica* (Fig. 2A) and *B. pertussis* (Fig 2B). Presence of DNase I in the culture medium at the time of inoculation resulted in a time-dependent effect on the ability of both the species to form biofilms. At the early time-point of 6h, addition of DNase I had no significant impact in inhibiting biofilm growth (Fig. 2). In contrast, at 12h or later of biofilm growth, DNase I significantly impaired the ability of both RB50 and Bp536 to form biofilms. These results suggest that while DNase I does not impede initial steps of biofilm development, it inhibits the steps of biofilm development that occur after 6h.

**Figure 3:**
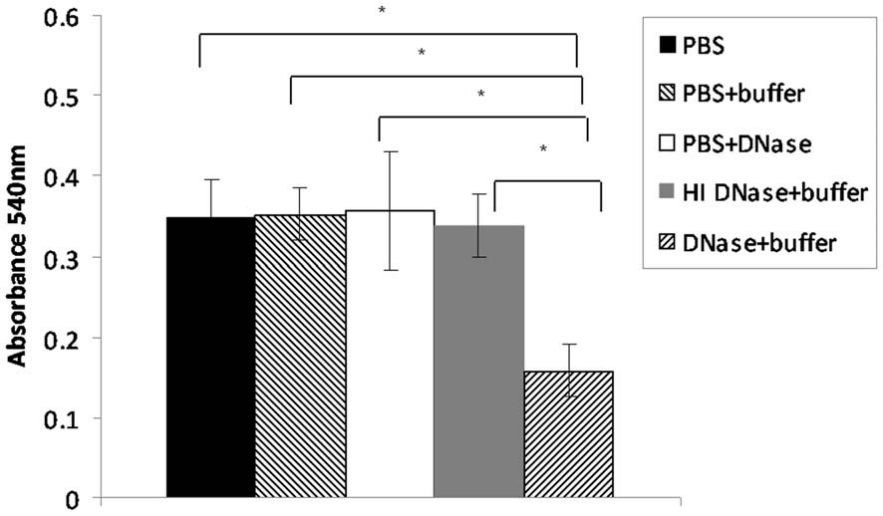
DNase I leads to the disruption of established *Bordetella* biofilms grown in microtitre plates. Preformed 48h RB50 biofilms grown in 96 well plates were rinsed with PBS followed by incubation with PBS, PBS and reaction buffer, PBS and DNase I, PBS and heat inactivated (HI) DNase I, or PBS with reaction buffer and DNase I. Biofilm formation was then quantitated via crystal violet staining. Each point is an average of at least 6 wells, and error bars indicate the standard deviation. Asterisks designate a value of *P*<0.05 (students *t*-test).

We performed several control experiments to ensure that the observed inhibitory effect was due to DNase I and not due to contaminating factors. Highly purified DNase I from another vendor (See Materials and Methods) resulted in similar levels of inhibition (Fig. S1). Incubation with either the DNase I reaction buffer (Fig. 2, black bars) or the heat inactivated enzyme did not result in any significant effect on biofilm formation (Fig. 3). The observed inhibition was also not due to a general bactericidal or bacteriostatic effect of DNase I, since the wild type strain showed similar growth in batch culture irrespective of the addition of DNase I (Fig. S2).

To determine whether the inhibitory effect of DNA on biofilm formation is reversible, we cultured RB50 in the presence of DNase I for 24h, washed with PBS to remove the enzyme followed by incubation in the culture medium for another 24h. As shown in Fig. S3, an increase in the levels of crystal violet staining was observed, suggesting that biofilm formation can progress after the removal of DNase I. We also determined that the DNase I used in these experiments was enzymatically active after 48h of incubation at 37°C, suggesting that biofilm formation at the extended time points involves an eDNA-dependent mechanism (Fig. S4).

### Detachment of preformed mature biofilms by DNase I

To further characterize the role of eDNA in *Bordetella* biofilms, specifically its contribution to the development of late stage biofilms, we determined if DNase I can disrupt statically established mature biofilms.

### Microtitre biofilm assays

Fig. 3 shows 48h biofilms formed in microtitre plates treated for 2h with DNase I. It is clear that incubation of these surface-associated biofilms with DNase I resuspended in the reaction buffer caused significant detachment. In control experiments, incubation with PBS alone, with PBS containing the DNase I reaction buffer, or with DNase I resuspended in PBS did not result in any significant removal of these biofilms from the surface. The failure of DNase I in PBS to have any effect on biofilm disruption is consistent with the requirement of cations for optimum activity of this enzyme.

### Microscopic analyses

**Figure 4:**
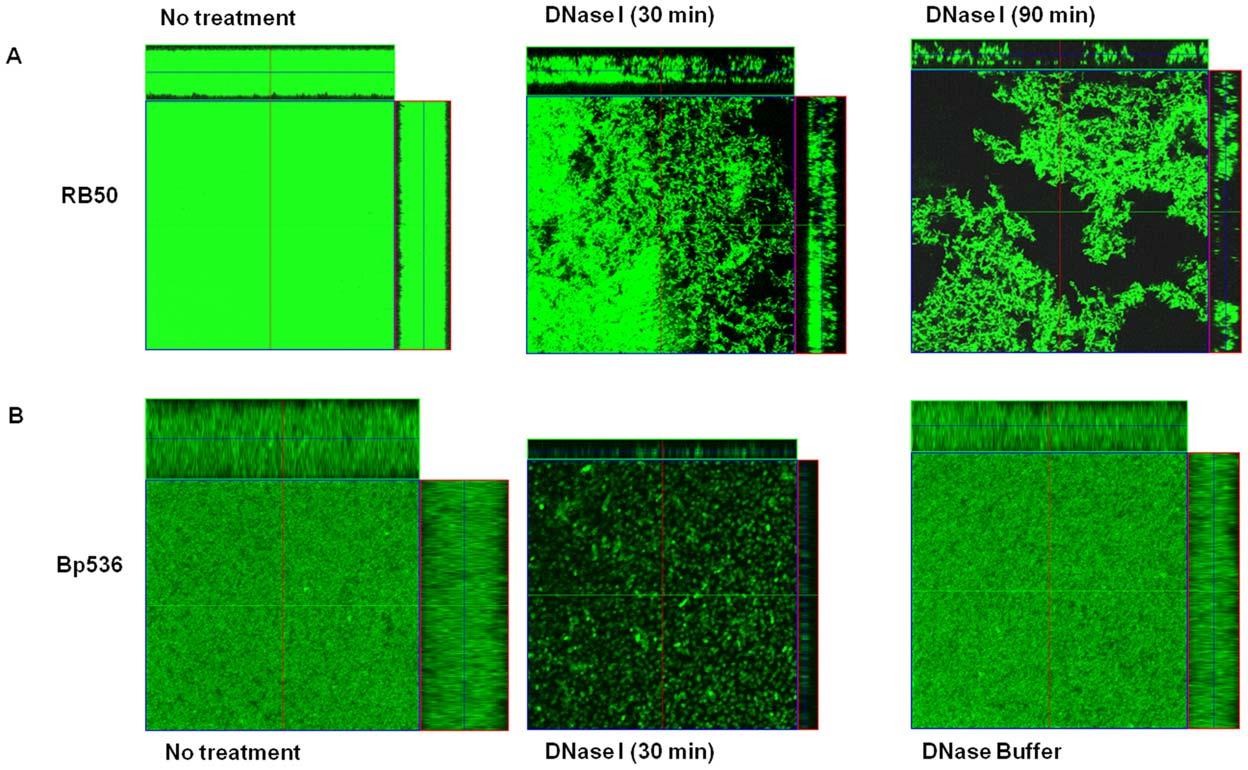
DNase I leads to the disruption of established *Bordetella* biofilms grown on glass coverslips under static conditions. Biofilms were grown on glass coverslips for 48h for RB50 (A) and 96 h for Bp536 (B). The coverslips were gently rinsed followed by treatment with DNase I for either 30min or 90min. The cells were tagged with GFP and thus are green. For each micrograph, the middle panel represents the *x-y* plane, and the adjacent top and side panels represent the *x-z* and *y-z* planes, respectively. The images of a biofilm not treated with DNase I and treated only with DNase I buffer are also depicted. CLSM was utilized to image the biofilms.

To visualize the effect of DNase I on impacting biofilm structure, we continued this experiment with biofilms of GFP-expressing cells formed on glass coverslips in biphasic cultures. For *B. bronchiseptica*, the biofilms were grown for 48h followed by incubation with or without DNase I for either 30 or 90 minutes. In the absence of DNase I, the glass coverslip was extensively colonized resulting in the visualization of a thick layer of cells at the air-liquid interface (Fig. 4A). DNase I treatment for 30 min led to the dissolution of the preformed bacterial films and the cells existed in patchy localized clusters (Fig. 4A). On longer incubation with DNase I, we found that large areas of the coverslips were essentially devoid of bacterial cells, suggesting significant detachment of the biofilm biomass (Fig. 4A).

Because *B. pertussis* has a longer generation time [38], we treated *B. pertussis* biofilms established for 4 days with DNase I. We have previously observed that at this time point, *B. pertussis* biofilms have defined architecture and reach structural maturity [15]. Treatment of preformed *B. pertussis* biofilms with DNase I resulted in dissolution of the biofilm (Fig 4B). Similar to that observed in Fig. 3, incubation with DNase I reaction buffer only did not have any visible effect on the formation of these biofilms (Fig. 4B). These results suggest that DNase I leads to detachment of preformed biofilms of both *B. bronchiseptica* and *B. pertussis*. The effect of DNase I on established biofilms was further quantified with the COMSTAT software package. This analysis revealed that DNase I treatment resulted in a drastic reduction in both the average and maximal thickness of the biofilms formed by both *B. bronchiseptica* and *B. pertussis* (Table 1).

**Table 1:** COMSTAT analysis of statically grown *Bordetella* biofilms treated with DNase I.

	Average Thickness (µm)	Maximum Thickness (µm)
RB50 No Treatment	27.3	28.8
RB50 30 min DNase I	4.5	19.2
RB50 90 min DNase I	5.6	13.6
Bp536 No Treatment	15.7	17.0
Bp536 30 min DNase I	0.05	3.0
RB50 DNase I Buffer Alone	22.9	26.1

Taken together, results presented so far suggest that in the presence of DNase I, while bacteria were able to attach to the surface, they failed to form stable biofilms. These results also indicate that eDNA is a crucial structural component of the *Bordetella* biofilm matrix and its removal through DNase I treatment disrupts the integrity of established biofilm.

### Extracellular DNA is crucial for biofilm maturation under hydrodynamic conditions

Biofilms grown under static conditions often lack or have less of the characteristic architecture compared to those grown under hydrodynamic conditions. Previously, we have shown that mature *Bordetella* biofilms formed under shear conditions appear in the form of pillars and towers with distinctive water channels [15], [17]. Thus, we examined whether eDNA is critical for the stability of structured biofilms formed under hydrodynamic conditions. GFP expressing *B. bronchiseptica* cells were inoculated into chambered flow cells and imaged using CLSM at an early (6h), middle (72h) and at late (120h) time-points during biofilm development. The presence of DNA in the biofilms was tracked using DDAO.

**Figure 5:**
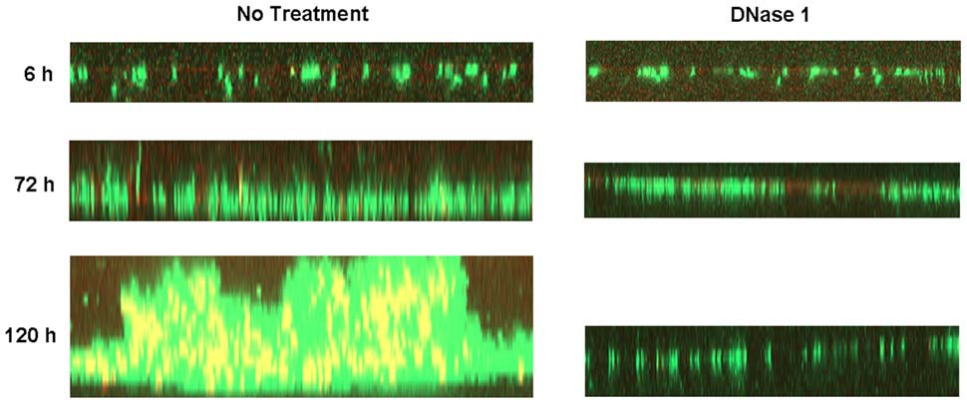
Susceptibility of flow cell biofilms to DNase I. Representative z-reconstructions of RB50 biofilms grown under flow conditions for 6, 72, or 120h and imaged using CLSM for live GFP expressing cells (green) and eDNA stained with DDAO (red or yellow with co-localization). The image of untreated biofilms (left panels) were taken immediately prior to incubation with DNase I and the images of same biofilms treated with DNase I for 1.5h (left panels). Images shown here are representative of two independent experiments.

At the early time point of biofilm formation, bacteria existed mainly in diffuse thin patches with large areas of coverslips remaining free of bacterial cells. At this time point, we were unable to detect significant levels of DNA (Fig. 5). At 72h, the biofilm increased in thickness and density with the emergence of some structural features in the form of thin closely clustered spikes or pillars. At this stage of biofilm formation, DNA was present in low but detectable levels. Dramatically at 120h of growth, the bacterial cells displayed attributes of highly structured biofilms with appearance of thick dome shaped pillar structures and water channels. Staining with DDAO revealed that this biofilm contained large amounts of DNA as indicated by yellow staining (Fig. 5). Unlike that observed with other bacteria, DNA did not appear to be localized at specific regions of the dome shaped biofilm structure [34], [36]. Instead DNA was found to be distributed throughout the biofilm. Note that these images are Z-reconstructions of multiple frames of the biofilms formed in the flow cell. Due to the compression and overlay of multiple images, colocalization of the red and green stains will occur. Thus, the yellow staining is indicative of eDNA closely associated with the GFP expressing bacteria.

Immediately after initial imaging of the bacterial cells and DNA, biofilms formed at the different time-points were treated with DNase I and then re-imaged. As shown in Fig. 5, upon DNase I treatment, there was very little effect on the biofilms formed at 6h, further confirming that eDNA plays only a minor role in the initiation of biofilm development. COMSTAT analysis also confirmed that there were no obvious differences between the DNase I treated and mock treated 6h biofilms, since for both the samples the average and maximum thickness was approximately 2.0 µm and 11 µm, respectively. On treatment of 72h biofilms with DNase I, we found that many areas of the coverslips were unoccupied suggesting surface detachment of bacterial cells. For the remaining biofilm that was resistant to DNase I treatment, the spike-like structures observed at this time point were reduced in height and were well separated. Strikingly, 120h biofilms were severely disrupted by DNase I leading to a complete dissolution of the architecture and integrity of the biofilms. Only a few regions of the coverslip were observed to have attached bacteria with no apparent biofilm architecture. As determined by COMSTAT analyses, the average biofilm thickness was reduced from 6.5 µm to 4.0 µm and the maximal thickness was reduced from 16.0 µm to 6.0 µm. In combination, these results suggest that DNA is a central component that promotes the stability of *Bordetella* biofilms formed on artificial surfaces.

### DNase I treatment disrupts *Bordetella* biofilms formed in the mouse nose

**Figure 6:**
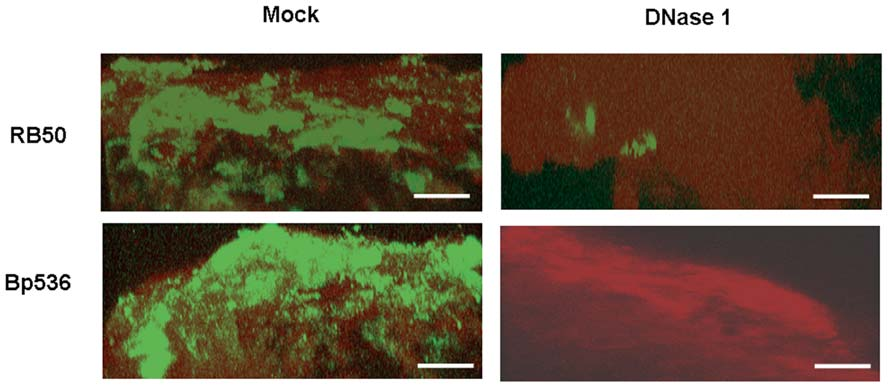
DNase I disrupts established biofilms of *B. bronchiseptica* and *B. pertussis* formed in the mouse respiratory tract. CLSM images of biofilms harvested from mouse nose 15 and 19 days postinoculation with RB50 (top) or Bp536 (bottom), respectively. The harvested nasal septum was excised into two equal parts and incubated either with DNase I buffer (Mock, left panels) or with DNase I (right panels) before processing for staining as described in the Materials and Methods. Green staining depicts *Bordetella* biofilms formed on top of the host epithelium, which is stained red.

There is no information available on whether eDNA contributes to the structural stability of *in vivo* biofilms formed in mammalian hosts. Extracellular DNA was recently visualized in *H. influenzae* biofilms formed in the chinchilla middle ear [39]. Although treatment with DNase I led to the removal of DNA, mature biofilms containing viable bacteria were still visible [39]. Because DNase I disrupted highly-structured *Bordetella* biofilms formed on abiotic surfaces, we hypothesized that eDNA will also be critical for structural stability of nasal biofilms. Thus, we determined whether DNase I treatment disrupted biofilms formed on the nasal septum of a mouse intranasally inoculated with *Bordetella*. Nasal septa were harvested from mice infected with *B. bronchiseptica* for 15 days or *B. pertussis* for 19 days and treated with either DNase I or with the DNase I reaction buffer. *Bordetella* biofilms formed on these tissues were then visualized by CSLM as described in the Materials and Methods. Microscopic analysis revealed the presence of bacteria in the form of scattered mat like structures on nasal epithelia represented by the green staining bacteria on the surfaces of the red staining epithelium (Fig. 6). Treatment with DNase I resulted in a fluorescent image where the vast majority of staining indicative of only the nasal epithelia was visible. Only a few patches of green fluorescence, indicative of bacterial cells, were observed for RB50, whereas no green fluorescence was observed for Bp536 (Fig. 6).

An alternate explanation for the above outcome is that treatment with DNase I is somehow interfering with the recognition of the bacterial strains by the rat anti-*Bordetella* serum, resulting in the inability to visualize the bacterial cells. To address this, we treated *in vitro* grown strains with DNase I followed by immunofluorescence staining. Microscopic observations revealed that there was no apparent reduction in the fluorescence of the bacterial cells as a result of DNase I treatment (data not shown).

**Figure 7:**
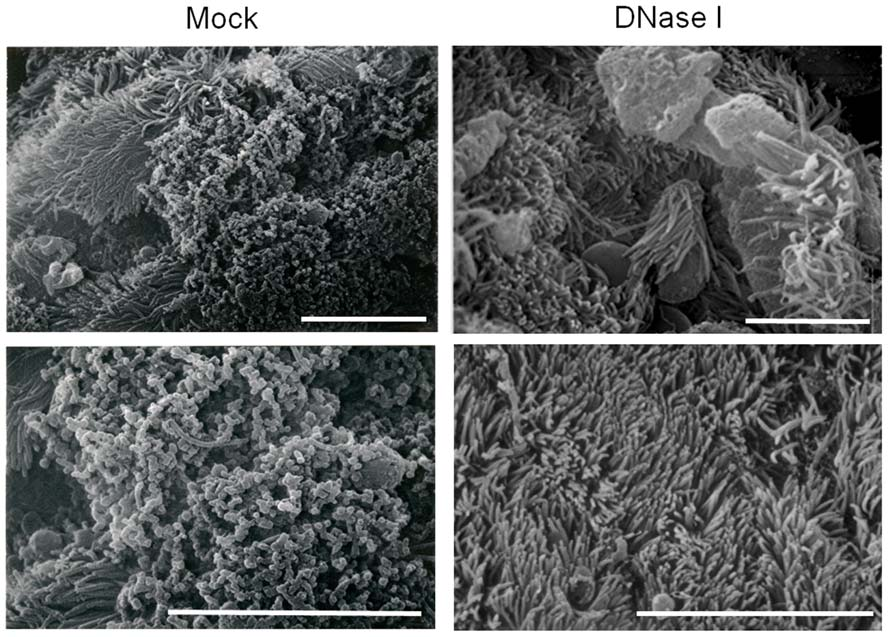
Scanning electron microscopy of mock treated (left) or DNase I treated *Bordetella* biofilms formed in the mouse nose. Nasal septa were harvested from mice 15 days post-inoculation, excised into two equal parts, treated with either the DNase I buffer (Mock, left panels) or DNase I (right) followed by processing for SEM as described in the Materials and Methods.

In addition to CSLM, we also utilized scanning electron microscopy (SEM) to examine the contribution of eDNA to the stability of *in vivo* biofilms. Mock treated nasal septa revealed the presence of thick mats of *B. bronchiseptica* encased in matrix material which appeared to completely obscure the underlying ciliated epithelium (Fig. 7). Treatment with DNase I resulted in dispersal of the biofilm exposing the ciliated epithelium underneath (Fig. 7). We also determined if incubation with DNase I affected nasal morphology. When observed microscopically, no gross and overt morphological differences were observed between nasal septa from naïve mice that were incubated with either PBS or DNase I. (Fig. S5). Taken together, results from Figs. 6 and 7 strongly suggest that treatment with DNase I leads to the detachment of respiratory tract biofilms of both *B. bronchiseptica* and *B. pertussis*.

## Discussion

Research on *Bordetella* biofilm development and macromolecules that constitute the biofilm matrix is at a very early stage. Specifically, the function of eDNA in the growth and the establishment of *Bordetella* biofilms is not known. One of the defining characteristics of bacterial biofilms is the presence of an extracellular matrix composed primarily of extracellular polysaccharides, embedded proteins and DNA, which provide the structural scaffold critical for stability of these biofilms [25], [26], [40]. Our study has uncovered a critical function for eDNA as an important structural component for *Bordetella* biofilm formation. Strikingly, we found that pre-established late-stage biofilms formed under static conditions and mature highly structured biofilms formed under shear forces were quite efficiently disrupted by DNase I.

The structural role of eDNA in promoting biofilm stability is highly variable and is dependent on the bacterial species, growth conditions and age of the biofilm. The relative abundance of DNA in the biofilm matrix compared to the other polymers does not appear to be a critical stability determining factor. For reference strains of *P. aeruginosa*, while extracellular DNA was the most abundant matrix polymer in a mature biofilm, exopolysaccharides appeared to be the most critical structural component [41]. Young and relatively unstructured biofilms of *P. aeruginosa* are dissolved easily by DNase I whereas mature and structured biofilms are resistant or only marginally affected [42]. For *Neisseria meningitidis*, only initial biofilm formation is supported by eDNA [43]. DNase I appears to have more pronounced effect on mature biofilms formed by Gram positive bacteria. For *Staphylococcus epidermidis* it has been shown that the effect of DNase I on biofilm dispersion decreased with time and DNase I had a minor effect on biofilms that were older than 12h [44]. Older biofilms of *S. aureus* initiated with high inoculum, and mature biofilms of *Enterococcus faecalis* and *Listeria monocytogenes* are sensitive to DNase I treatment to varying degrees [35], [36], [37]. Our finding that DNase I can disrupt a mature multicellular *Bordetella* biofilm grown under both static and hydrodynamic conditions has not been reported previously for any other Gram negative bacteria. This suggests that unlike many other Gram negative bacteria, *Bordetella* utilizes eDNA as a key component to confer structural stability to biofilms. We have previously shown that the Bps exopolysaccharide, a component of the *Bordetella* biofilm matrix, is critical for the establishment of mature and structured biofilm [15], [17]. It appears the formation of structured biofilms in *Bordetella* is dependent on at least two macromolecules, Bps and eDNA. Based on protein homology of biosynthetic components and antibody cross-reactivity, Bps is similar to the poly-β-1,6-N-acetylglucosamine polysaccharides synthesized by many pathogenic bacteria. These polysaccharides in other species are partially deacetylated thereby imparting a net positive charge on the bacterial surface [45], [46], [47]. The overall positive charge of these polysaccharides may facilitate interactions with the negatively charged eDNA. We hypothesize that by forming a physical complex, eDNA and Bps promote the formation of mature biofilms of *Bordetella* spp. The interaction of DNA with polysaccharides has previously been demonstrated. The β-1,3-glucans polysaccharides specifically interact with certain polynucleotides to form triple-stranded and helical macromolecular complexes [48]. Similarly eDNA from *Caulobacter crescentus* binds to the holdfast polysaccharide, which is composed in part of β-1,4-N-acetylglucosamine polysaccharides. Interestingly, this interaction was demonstrated to prevent the settlement of cells into biofilms and to promote dispersal [31].

The mechanisms of the release and accumulation of eDNA in biofilms are poorly understood. Autolysis of cells in microcolonies has been hypothesized to mediate DNA release [49], [50]. In *E. faecalis*, regulated autolysis by the action of two proteases results in release of eDNA in biofilm [51]. In *Staphylococcus aureus*, a finely tuned holin/antiholin system is thought to mediate cell lysis and programmed cell death. This system is comprised of the *cidAB* and *lrgAB* operons which encode for proteins that are analogous to bacteriophage holins and antiholins, respectively [49], [52]. It has been proposed that by differential expression of the Cid and the Lrg proteins, cell lysis and subsequent release of eDNA is controlled during biofilm development [49], [50], [53]. Our data show that the release of DNA into biofilms is conserved in both *B. bronchiseptica* and *B. pertussis*. *Bordetella* spp. harbor genes homologous to *cidA* and *cidB* of *S. aureus* [49]. *B. bronchiseptica* is considered the evolutionary progenitor of *B. pertussis*. Despite this evolutionary relationship, these two species differ greatly in genome size and gene expression patterns. *B. pertussis* has lost close to 1 Mb of the genome and contains a large number of pseudogenes, many of which have been inactivated by insertion elements, in-frame stop codons, and frameshift mutations [54]. Thus, it is reasonable to speculate that the *cid* homologues of *B. pertussis* will be important for one or more of the virulence characteristics and growth in hosts. Our future efforts will be directed towards deciphering the function of the *Bordetella cidAB* homologues in eDNA release, biofilm development and virulence.

While it is clear that eDNA plays a critical role in maintaining the architectural integrity of bacterial biofilms formed on abiotic surfaces under laboratory conditions, its contribution to biofilm stability *in vivo* remains largely unexplored. A network of extracellular DNA fibers have been visualized in *H. influenzae* biofilms formed in the chinchilla middle ear [39], [55]. In one of these studies, even though extracellular DNA was removed on treatment with DNase I, viable bacteria were still visible in these biofilms [39]. Our attempts to detect DNA in the mouse nasopharynx have been unsuccessful. Utilization of stains routinely used for DNA staining resulted in staining of the entire nasal epithelium, making it difficult to detect the presence of potential DNA fibres and the *Bordetella* cells. In this report, we have obtained evidence of DNA being responsible for biofilm stability in mammalian hosts. We found that DNase I treatment of the mouse nasal septum led to a drastic dissolution of the resident biofilms suggesting that extracellular DNA is a critical cell-cell interconnecting macromolecule in respiratory tract biofilms.

To conclude, this study provides further insights into the mechanisms responsible for biofilm development in *Bordetella*. Our results strongly support the role of eDNA in maintaining biofilm stability and document for the first time the ability of DNase I to degrade biofilms formed in an animal model of bacterial virulence. We propose that DNase I represents an accessory option along with the already approved immunization regimens for the management and treatment of *Bordetella*-associated infections in both humans and animals [56].

## Supporting Information

Figure S1 **DNase I from different vendors disrupts preformed** ***Bordetella*** **biofilms.** 48 h *B. bronchiseptica* (RB50) biofilms formed in 96 well plates were treated with DNase I (100 Kuntz units/ ml) from Promega or Sigma for 1 h. The biofilm was then stained with crystal violet for quantification at O.D._540_. Error bars represent the standard deviation. Asterisks designate a value of *P*<0.05 (students *t*-test).
(TIF)

Figure S2 **Incubation with DNase I does not alter the growth kinetics of** ***B. bronchiseptica*** **.** SS broth was supplemented with either the DNase I buffer or the buffer plus DNase I (40 Kuntz units/ ml) followed by inoculation of RB50 at an O.D._600_ of 0.1. The culture tubes were incubated at 37°C with shaking. At different time points, the O.D._600_ was measured for each culture.
(TIF)

Figure S3 **Reversibility of DNase I biofilm disruption.** RB50 was grown in 96 well plates in SS medium with either DNase I resuspended in the reaction buffer or in the reaction buffer alone (Mock). Shown as controls are biofilms that were treated with DNase I for 24h or for 48 h. For one set of DNase I treated biofilms, the wells were washed with PBS after 24 h followed by incubation in SS broth for an additional 24 h (24 h DNase/wash). The biofilm were stained with crystal violet for quantification at O.D._540_. Error bars represent the standard deviation. Asterisks designate a value of *P*<0.05 (students *t*-test).
(TIF)

Figure S4 **DNase I stability.** DNase I from Sigma or from Promega was incubated at 37°C for 48 h in the reaction buffer. Genomic DNA from RB50 was then treated with either the DNase I buffer alone (Mock) or with the pre-incubated DNase I samples at 37°C for 3 h. Samples were run on a 1% agarose gel to determine if the DNA had been digested by DNase I.
(TIF)

Figure S5 **Incubation with DNase I does not alter the morphology of the mouse nasal septum as observed by SEM.** Nasal septa harvested from naïve mice were suspended in PBS followed by treatment at 37°C for 2 h with either the DNase I buffer (Mock, left panel) or with DNase I resuspended in the DNase I buffer (DNase I, right panel) followed by visualization with SEM. Bar, 10µm.
(TIF)
