# Supplementary figures and images for: Extracellular DNA Is Essential for Maintaining Bordetella Biofilm Integrity on Abiotic Surfaces and in the Upper Respiratory Tract of Mice
Source: PLoS One. 2011 Feb 11;6(2):e16861. doi: 10.1371/journal.pone.0016861 (PMC3037945; doi:10.1371/journal.pone.0016861)

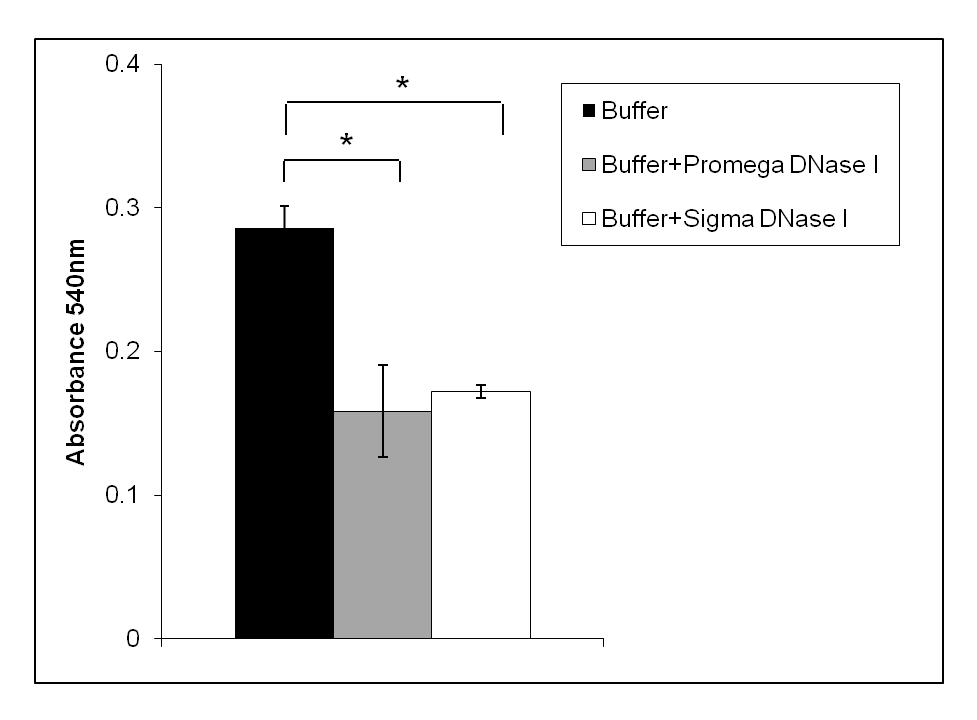

Supplement: Figure S1 — DNase I from different vendors disrupts preformed Bordetella biofilms. 48 h B. bronchiseptica (RB50) biofilms formed in 96 well plates were treated with DNase I (100 Kuntz units/ ml) from Promega or Sigma for 1 h. The biofilm was then stained with crystal violet for quantification at O.D.540. Error bars represent the standard deviation. Asterisks designate a value of P<0.05 (students t-test). (TIF) [file pone.0016861.s001.tif]

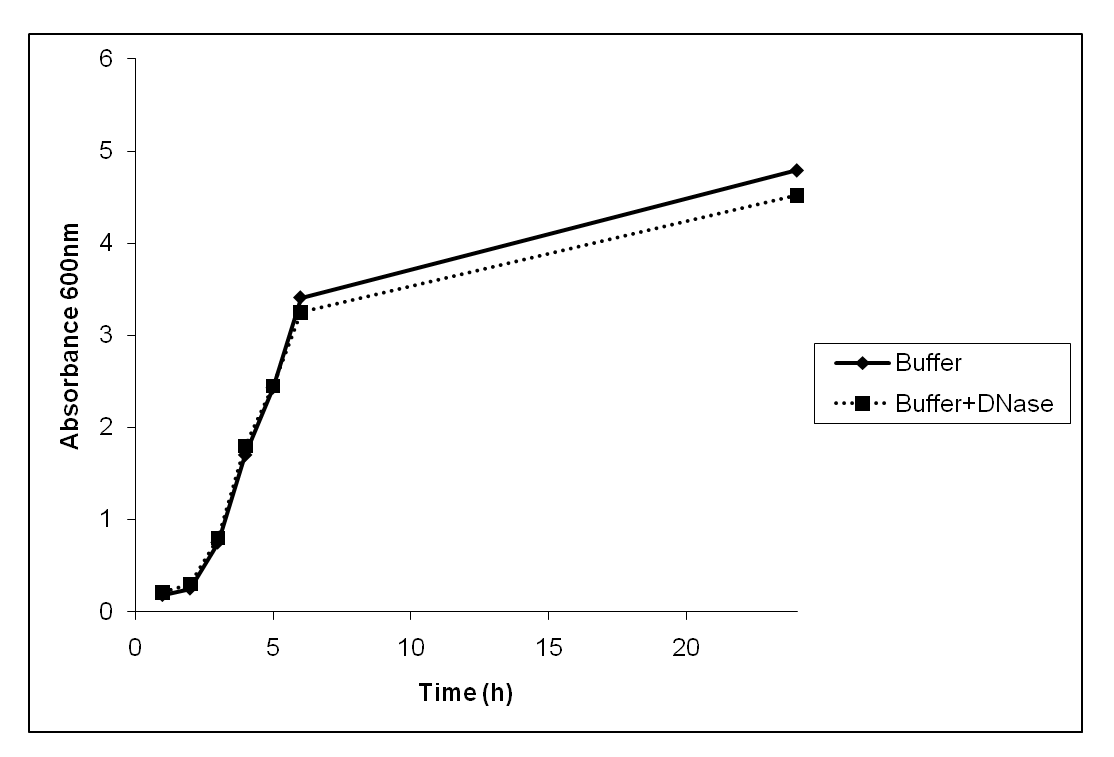

Supplement: Figure S2 — Incubation with DNase I does not alter the growth kinetics of B. bronchiseptica . SS broth was supplemented with either the DNase I buffer or the buffer plus DNase I (40 Kuntz units/ ml) followed by inoculation of RB50 at an O.D.600 of 0.1. The culture tubes were incubated at 37°C with shaking. At different time points, the O.D.600 was measured for each culture. (TIF) [file pone.0016861.s002.tif]

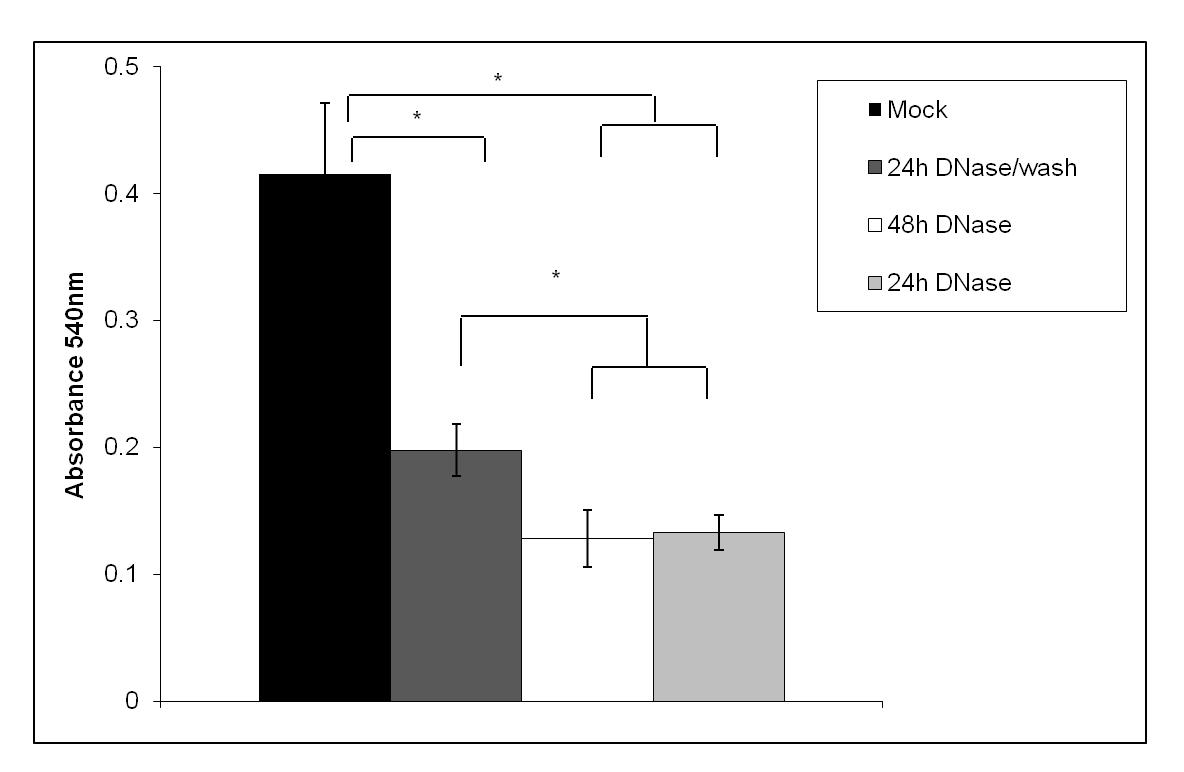

Supplement: Figure S3 — Reversibility of DNase I biofilm disruption. RB50 was grown in 96 well plates in SS medium with either DNase I resuspended in the reaction buffer or in the reaction buffer alone (Mock). Shown as controls are biofilms that were treated with DNase I for 24h or for 48 h. For one set of DNase I treated biofilms, the wells were washed with PBS after 24 h followed by incubation in SS broth for an additional 24 h (24 h DNase/wash). The biofilm were stained with crystal violet for quantification at O.D.540. Error bars represent the standard deviation. Asterisks designate a value of P<0.05 (students t-test). (TIF) [file pone.0016861.s003.tif]

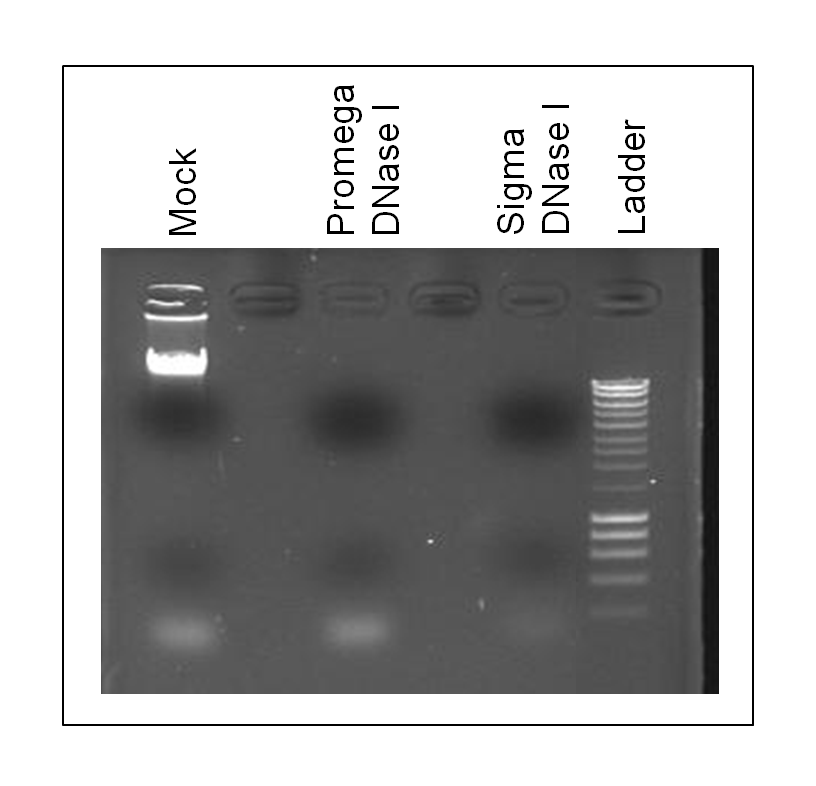

Supplement: Figure S4 — DNase I stability. DNase I from Sigma or from Promega was incubated at 37°C for 48 h in the reaction buffer. Genomic DNA from RB50 was then treated with either the DNase I buffer alone (Mock) or with the pre-incubated DNase I samples at 37°C for 3 h. Samples were run on a 1% agarose gel to determine if the DNA had been digested by DNase I. (TIF) [file pone.0016861.s004.tif]

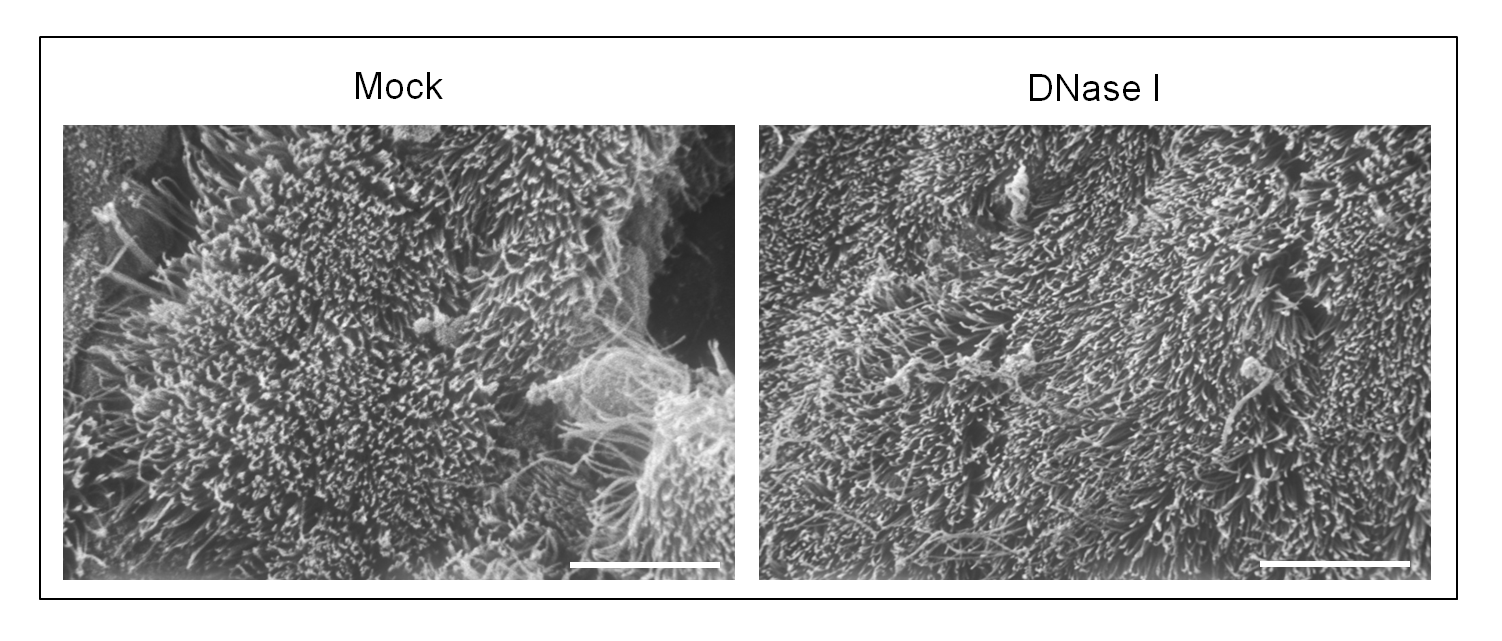

Supplement: Figure S5 — Incubation with DNase I does not alter the morphology of the mouse nasal septum as observed by SEM. Nasal septa harvested from naïve mice were suspended in PBS followed by treatment at 37°C for 2 h with either the DNase I buffer (Mock, left panel) or with DNase I resuspended in the DNase I buffer (DNase I, right panel) followed by visualization with SEM. Bar, 10µm. (TIF) [file pone.0016861.s005.tif]
